# Cerebral dopamine neurotrophic factor protects and repairs dopamine neurons by novel mechanism

**DOI:** 10.1038/s41380-021-01394-6

**Published:** 2021-12-14

**Authors:** Päivi Lindholm, Mart Saarma

**Affiliations:** Institute of Biotechnology, Helsinki Institute of Life Science, University of Helsinki, FI-00014 Helsinki, Finland

**Keywords:** Molecular biology, Neuroscience

## Abstract

Midbrain dopamine neurons deteriorate in Parkinson’s disease (PD) that is a progressive neurodegenerative movement disorder. No cure is available that would stop the dopaminergic decline or restore function of injured neurons in PD. Neurotrophic factors (NTFs), e.g., glial cell line-derived neurotrophic factor (GDNF) are small, secreted proteins that promote neuron survival during mammalian development and regulate adult neuronal plasticity, and they are studied as potential therapeutic agents for the treatment of neurodegenerative diseases. However, results from clinical trials of GDNF and related NTF neurturin (NRTN) in PD have been modest so far. In this review, we focus on cerebral dopamine neurotrophic factor (CDNF), an unconventional neurotrophic protein. CDNF delivered to the brain parenchyma protects and restores dopamine neurons in animal models of PD. In a recent Phase I-II clinical trial CDNF was found safe and well tolerated. CDNF deletion in mice led to age-dependent functional changes in the brain dopaminergic system and loss of enteric neurons resulting in slower gastrointestinal motility. These defects in *Cdnf*^−/−^ mice intriguingly resemble deficiencies observed in early stage PD. Different from classical NTFs, CDNF can function both as an extracellular trophic factor and as an intracellular, endoplasmic reticulum (ER) luminal protein that protects neurons and other cell types against ER stress. Similarly to the homologous mesencephalic astrocyte-derived neurotrophic factor (MANF), CDNF is able to regulate ER stress-induced unfolded protein response (UPR) signaling and promote protein homeostasis in the ER. Since ER stress is thought to be one of the pathophysiological mechanisms contributing to the dopaminergic degeneration in PD, CDNF, and its small-molecule derivatives that are under development may provide useful tools for experimental medicine and future therapies for the treatment of PD and other neurodegenerative protein-misfolding diseases.

## Introduction

Increased life expectancy and a growing aging population are leading to an increase in the incidence of age-related diseases, including Parkinson’s disease (PD) which affects 1% of population over 60 years of age [1], and with more than 6 million people diagnosed with PD globally [2]. PD is a progressing neurodegenerative movement disorder, in which midbrain dopamine (DA) neurons in the substantia nigra (SN) degenerate and die. Major motor symptoms of PD are slowness of movement, resting tremor, rigidity, and postural instability that appear when there is about 30% loss of DA neurons in the SN and 50–60% reduction in striatal DA axon terminals [3]. Patients with PD also suffer from non-motor symptoms, including constipation, hyposmia, depression, lack of motivation, sleep disorders, and cognitive decline that significantly decrease quality of life [4, 5].

Although a few toxins and genetic mutations are known to cause PD, the etiology is unknown in majority of cases. While precise mechanisms of DA neuron death are unclear, increasing body of evidence suggests that protein aggregation, mitochondrial dysfunction, inflammation, and reduced growth factor levels are involved in the molecular pathogenesis of PD [6, 7]. Aggregation of misfolded α-synuclein (αSyn), a major component of intraneuronal Lewy bodies, may possibly cause endoplasmic reticulum (ER) stress in DA neurons leading to neuronal death [8, 9]. Lewy body pathology can be widespread in the central nervous system (CNS) as well as in the peripheral nervous system (PNS) including the enteric nervous system (ENS) [10]. The non-motor symptoms of PD can be related to the dysfunction of DA and other neurotransmitter systems, such as the noradrenergic and cholinergic systems [4]. However, the neuropathological mechanisms behind the non-motor symptoms are largely unknown.

**Fig. 1:**
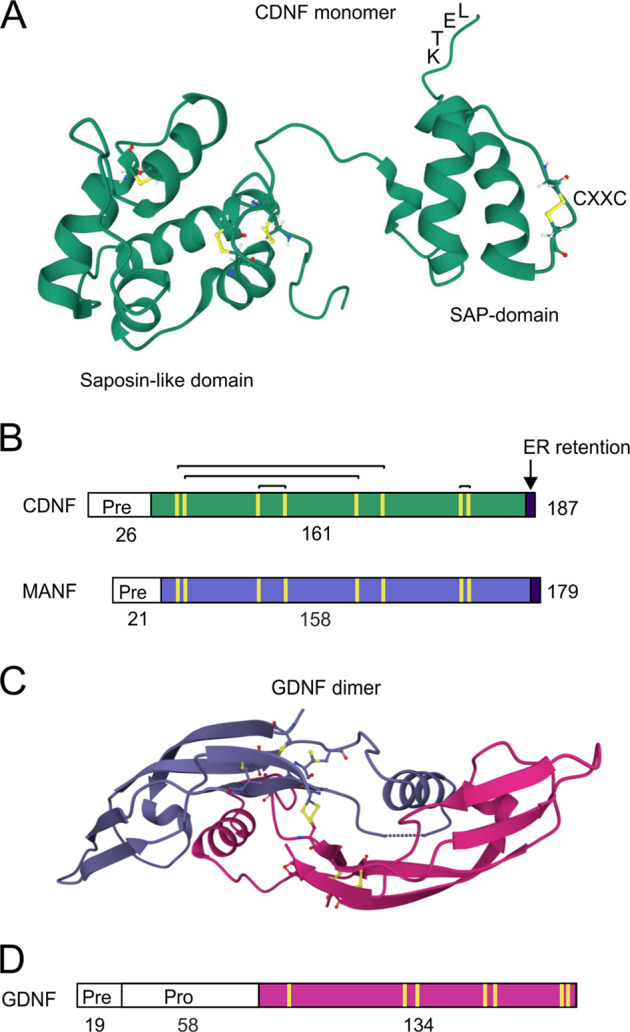
Structural features of human CDNF, MANF, and GDNF proteins. **A** CDNF (PDB ID: 4BIT [30]) is a monomeric protein. It has an amino-terminal saposin-like domain that may mediate interaction with lipids, and a carboxy-terminal SAP (SAF-A/B, Acinus, and PIAS) domain. The CXXC motif (CRAC and CKGC in CDNF and MANF, respectively) forming a cysteine bridge is located in the C-terminal domain. Cysteine bridges stabilizing the 3D structure are shown in yellow. An ER retention signal (KTEL) is in the C-terminus of CDNF. **B** Primary structure of CDNF and MANF. CDNF/MANF proteins have an N-terminal signal peptide directing them to the ER (Pre). Conserved cysteine residues in mature CDNF (green) and MANF (blue) are indicated as yellow bars, and disulfide bridges as black connecting lines. Human mature CDNF and MANF consist of 161 and 158 amino acid residues, respectively, and the amino acid identity between them is 59%. **C** Two GDNF (PDB ID: 1AGQ [126]) monomers (molecular mass 15 kDa; indicated in blue and red) are connected by an intermolecular disulfide bridge (in yellow) to form a homodimer. **D** GDNF primary structure contains a signal sequence (Pre) directing it to the secretory pathway, a pro-sequence that is enzymatically cleaved releasing mature GDNF (red) with seven conserved cysteines (in yellow). Number of amino acid residues is indicated.

Treatments are available that can improve motor symptoms of PD in most patients, but no disease-modifying therapy exists. Future therapies should include interventions that slow down or prevent the degeneration and death of DA neurons, regenerate the remaining DA neurons and increase their functional activity. They should also alleviate non-motor symptoms of PD. Neurotrophic factors (NTFs) hold great promise as drugs that could promote neuroprotection of DA neurons, and even have the capacity to regenerate them. NTFs are small, secreted proteins that promote neuronal survival, regulate development, function and maintenance of neurons, and advance neuronal recovery from injury [5, 11, 12]. Glial cell line-derived neurotrophic factor (GDNF) family ligands (GFLs) GDNF (Figs. 1C, D and 3A) and NRTN have been shown to be efficient in protecting DA neurons in rodent and non-human primate (NHP) models of PD, but have only shown modest effects in Phase II clinical trials in PD patients [5, 13, 14]. GDNF has not been shown to be neuroprotective in the rodent αSyn model of PD, where αSyn was overexpressed by viral vectors [15], but it in vitro and in vivo protects DA neurons from accumulation of misfolded αSyn [16]. Why have GDNF and NRTN given modest therapeutic effects in clinical trials so far? One of the reasons is that patient populations with advanced PD were treated in the Phase II clinical trials [17, 18]. Five years after clinical diagnosis, PD patients have almost no striatal dopaminergic fibers left and have pronounced loss of DA neuron cell bodies in the SN [19]. Another important aspect is limited diffusion of GDNF and NRTN in brain parenchyma that can decrease target engagement [5, 14]. A major limitation of NTF therapy is the requirement for their intracranial delivery using invasive brain stereotactic surgery, as NTF proteins do not cross the blood–brain barrier (BBB). In order to find out the real value of NTF therapy, several factors should be taken into consideration. Firstly, treatment should be started as soon as possible following the clinical diagnosis of PD. However, currently this is regulated by ethical considerations, which do not allow invasive surgery for the treatment of early stage PD patients. Secondly, gene technology and protein design can be used to improve the therapeutic and pharmacokinetic properties of NTFs. Thirdly, it is possible to search for new trophic factors and neurotrophic small molecules with better therapeutic properties.

We have discovered a protein with NTF properties, named cerebral dopamine neurotrophic factor (CDNF) [20], that together with the related mesencephalic astrocyte-derived neurotrophic factor (MANF, also known as ARMET) [21], form a novel evolutionarily conserved family of unconventional NTFs [22–27]. CDNF and MANF have neurotrophic properties but they otherwise dramatically differ from other known NTFs (Table 1). They have a unique structure, mode of action and they can promote cellular protein homeostasis by regulating ER stress, regulate inflammation and support neuron survival in animal models of PD [22–27]. Surprisingly, variants of CDNF can cross through the BBB thus opening a new possibility for a systemic administration of this neurotrophic drug [28]. In this review, we discuss the structure, cellular effects, biology, and therapeutic potential of CDNF. We also briefly introduce characteristic features of MANF in order to give an overview of CDNF/MANF protein family.

**Table 1:** General properties of CDNF, GDNF, and NRTN proteins.

	CDNF	GDNF	NRTN	References
Protein family	CDNF/MANF	TGF-β	TGF-β	[20, 123, 124]
Structure	Saposin-like domain and SAP-domain	Cystine knot	Cystine knot	[29–31, 123, 124]
Polypeptide	Pre-CDNF	Prepro-GDNF	Prepro-NRTN	[20, 123, 125]
Number of amino acids in mature protein	161	134	102	[20, 123, 124]
Active conformation	Monomer	Homodimer, disulfide-linked	Homodimer, disulfide-linked	[30, 126, 127]
Molecular mass	18 kDa	32 kDa	25 kDa	[20, 123, 124]
Calculated pI	7.7	9.44	9.01	[31, 128, 129]
Heparin binding	Weak	Strong	Very strong	[130, 131]
Diffusion in brain tissue	Good	Limited	Very limited	[84, 100, 132]
Solubility	Good	Good	Poor	[20, 133]
Stability	Good	Good	Good	[101, 134]
Inhibits cell death	Yes	Yes	Yes	[20, 135, 136]
Regulates UPR	Yes	?	?	[70, 121]

### CDNF and MANF are structurally unique proteins regulating ER homeostasis

The three-dimensional structures of mature CDNF and MANF proteins consist of a unique combination of two domains, an amino-terminal (N-terminal) saposin-like domain and a carboxy-terminal (C-terminal) SAF-A/B, Acinus, and PIAS (SAP) domain [29–31] (Fig. 1A). The domains are connected by a flexible linker region suggesting that they can perform separate functions [29–31]. Since saposin-like proteins usually interact with lipids or membranes, it is probable that the N-terminal domain mediates the CDNF/MANF interaction with lipids [31]. Indeed, MANF was shown to directly bind sulfoglycolipid 3-*O*-sulfogalactosylceramide (sulfatide) possibly via its N-terminal domain [32]. The C-terminal SAP-domain is important for the neuroprotective activity of MANF, since it can independently promote the survival of neurons in vitro [29]. In their primary structure, CDNF/MANF proteins have eight cysteine residues with conserved spacing, which are important for the protein fold (Fig. 1B). Three intramolecular disulfide bonds stabilize the saposin fold of the N-terminal domain and a fourth disulfide bond can be formed in a CXXC motif in the SAP-domain [31]. When the CXXC motif was mutated, neuroprotective activity of MANF was lost indicating that this motif is crucial for the biological activity of MANF [33]. At the very C-terminal end, CDNF and MANF have an ER retrieval sequence resembling the canonical lysine-aspartic acid-glutamic acid-leucine (KDEL) sequence preventing protein secretion from the ER [34, 35] (Figs. 1A, B and 3C). In support for the role of KDEL-receptors (KDEL-Rs) in regulating CDNF and MANF secretion, deletion of the C-terminal KDEL-like sequence increases their release from cells [33–37]. Human CDNF has potential sites for N-linked and O-linked glycosylation but glycosylation is not required for its secretion [20, 38].

**Fig. 2:**
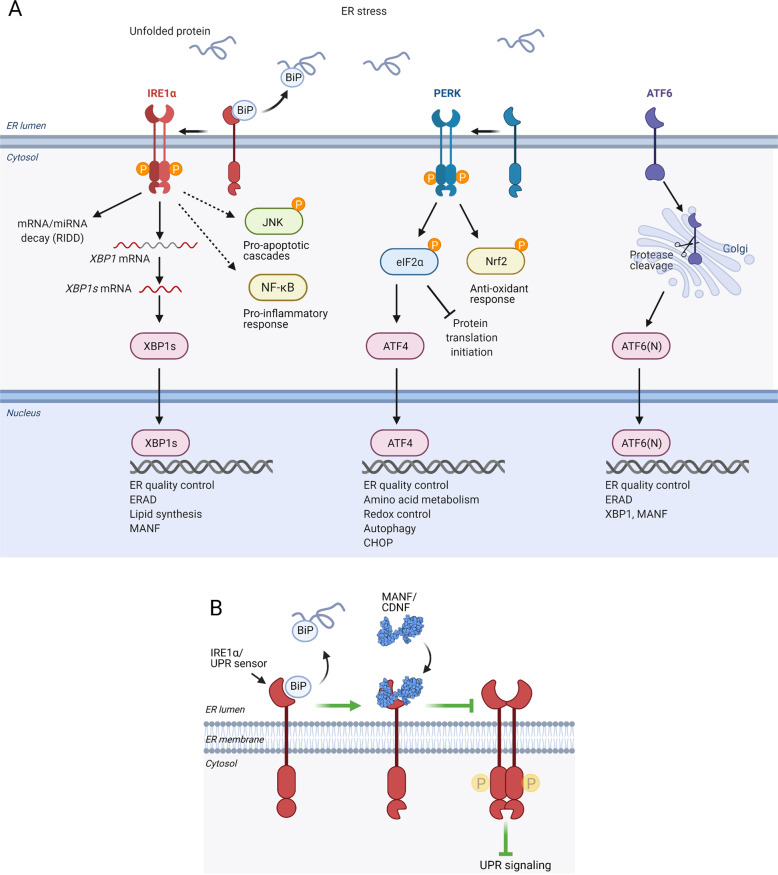
General cellular scheme of unfolded protein response (UPR), and a potential mechanism how MANF and CDNF are regulating UPR in the ER. **A** UPR is activated by ATF6, PERK, and IRE1α sensors located in the ER membrane of mammalian cells. In nonstressed conditions, ER chaperone BiP associates to the luminal domain of IRE1α, PERK, and ATF6 keeping them inactive. When unfolded proteins accumulate in the ER lumen causing ER stress, BiP is dissociated from the sensors, favoring activation of UPR. Unfolded proteins may also directly bind and activate IRE1α and PERK [137–139]. Upon activation, IRE1α forms homodimers and oligomers leading activation of its cytosolic kinase domain, *trans*-autophoshorylation and stimulation of its ribonuclease (RNase) activity. The active RNase domain of IRE1α removes an intron from *XBP1* mRNA leading to the expression of transcription factor XBP1s, which induces transcription of genes related to ER quality control, ER-associated degradation (ERAD), and lipid synthesis. The RNase of IRE1α may also degrade ER-targeted mRNAs and miRNAs through regulated IRE1-dependent decay (RIDD), thus decreasing protein folding demand. IRE1α can—via adapter TRAF2—regulate c-Jun N-terminal kinase (JNK) activation and apoptosis pathways, and NF-κB activation and pro-inflammatory signaling. Activated PERK phosphorylates α-subunit of eukaryotic initiation factor 2 (eIF2), leading to transient arrest of translation initiation and decreased general protein synthesis. PERK also phosphorylates transcription factor nuclear factor, erythroid 2-related factor 2 (NRF2) that regulates antioxidant response genes [140, 141]. Translation of ATF4 transcription factor is favored in conditions of limited eIF2α. ATF4 induces transcription of genes involved in protein folding, redox control, amino acid metabolism and autophagy. Under prolonged ER stress, ATF4 induces pro-apoptotic transcription factor CCAAT/enhancer-binding protein homologous protein (CHOP). Upon activation, ATF6 translocates to the Golgi where it is cleaved by endopeptidases, releasing ATF6(N) fragment that functions as a transcription factor. ATF6(N) induces expression of *XBP1* mRNA and components of ERAD. XBP1s and ATF6(N) can induce *MANF* expression. For in-depth discussion of UPR please see excellent reviews [142–144]. **B** MANF directly interacts with the ER luminal domain of UPR sensor IRE1α. MANF binding decreases ER stress-induced oligomerization and phosphorylation of IRE1α, leading to attenuation of UPR. BiP prevents MANF interaction with IRE1α, while MANF at physiological concentrations does not affect BiP–IRE1α interaction, which suggests that MANF binds and regulates the sensor activity after dissociation of BiP [65]. Similarly to MANF, CDNF may interact with a UPR sensor to regulate UPR.

In cells, CDNF and MANF reside mainly in the lumen of the ER [39, 40] where, especially MANF and likely CDNF have an important role in regulating of ER protein homeostasis and promoting cell survival under ER stress [23, 26]. ER stress is a condition where protein-folding capacity of the ER is overwhelmed resulting in accumulation of unfolded proteins in the lumen. It can be due to various physiological and pathological conditions, including increased demand of protein secretion, synthesis of mutant proteins, hypoxia, nutrient deprivation, or depletion of ER calcium. To overcome ER stress, an adaptive signal transduction pathway termed the unfolded protein response (UPR) is activated to restore ER protein homeostasis by increasing expression of chaperones to improve protein folding capacity, to attenuate translation to reduce protein folding load, and to enhance ER-associated protein degradation (ERAD) to remove misfolded proteins [41]. Three ER transmembrane proteins inositol-requiring enzyme 1α (IRE1α; also known as ERN1), protein kinase R-like ER kinase (PERK; also known as EIF2AK3) and activating transcription factor 6 (ATF6) function as sensors for disturbances in ER protein homeostasis in mammalian cells, and their activation induces UPR signaling [41–44] (Fig. 2A). If recovery of ER homeostasis fails, UPR can become chronic leading to apoptosis [45]. UPR has been associated with pathophysiology of several neurodegenerative protein-misfolding diseases, including PD [46–48].

Based on the structural homology between CDNF and MANF, we can hypothesize that their molecular mechanism of cytoprotective action has some similar features. Both CDNF and MANF are widely expressed in mammalian tissues although with differential levels [20, 49, 50] suggesting tissue-specific functions. The *MANF* promoter contains ER stress response elements recognized by UPR-induced transcription factors [39, 51, 52] and its expression is increased in ER stress-related conditions [39, 53–55]. Biological importance of endogenous MANF for the maintenance of ER protein homeostasis was demonstrated in conventional and pancreas-specific MANF knockout mice, where chronic UPR activation contributes to the loss of pancreatic insulin producing beta cell mass and development of diabetes mellitus-like condition [56, 57]. In cultured cells, silencing of MANF led to activation of UPR and increased susceptibility to ER stress-induced cell death [58]. UPR activation was also detected in *Caenorhabditis elegans* [59, 60] and *Drosophila melanogaster* [61] due to the loss of functional MANF. MANF interacts with an ER chaperone BiP [62, 63], and was shown to prolong BiP interaction with its clients thus promoting protein-folding homeostasis in the ER [64]. We recently observed that intracellular MANF is able to promote the survival of cultured neurons by a mechanism relying on the activity of either IRE1α or PERK pathways [63]. However, MANF interaction with BiP was not required for its neuroprotective activity [63]. Further studies demonstrated that MANF directly binds to the luminal domain of IRE1α [65]. MANF binding decreased ER stress-induced oligomerization and phosphorylation of IRE1α, leading to attenuation of UPR [65]. Under homeostatic conditions, BiP binds to the luminal domain of IRE1α, PERK, and ATF6 keeping them inactive, whereas in ER stress BiP is dissociated triggering the activation of UPR sensors [41]. MANF was shown to compete with BiP for the interaction with IRE1α suggesting that MANF is able to bind and regulate IRE1α activity only when BiP is dissociated, as is the case in ER stress [65]. Thus, IRE1α could act as MANF receptor in the ER and MANF, by moderating IRE1α activity could promote cell survival during ER stress [65] (Fig. 2B). The biological function of MANF in regulating ER protein homeostasis was further supported by protein–protein interaction studies suggesting that MANF is a member of a large multiprotein complex of ER chaperones [63]. A recent report demonstrated that MANF can function as a chaperone in the ER, although it does not show structural or sequence homologies to known chaperone families [66].

In ER stress-related disease models in vivo, expression of endogenous CDNF was reported to increase after cerebral or myocardial ischemia [67, 68]. In vitro, ER stress-inducing tunicamycin treatment increased CDNF expression in cardiomyocytes [69] but not in an osteosarcoma-derived cell line [58]. Thus, responsiveness of CDNF to ER stress may depend on cell type. However, intracellular CDNF was cytoprotective against ER stress and able to regulate UPR. Overexpression of CDNF alleviated ER stress-induced astrocyte damage, and attenuated the expression of ER stress-induced apoptotic proteins in neurons [70, 71]. What is more, CDNF overexpression may induce a mild adaptive conditioning UPR that prepares cells to encounter ER stress and protects cells in this way [70]. Whether CDNF can regulate UPR via binding to UPR sensors, similarly to the interaction of MANF and IRE1α, is unknown (Fig. 2B).

Although CDNF and MANF are largely retained in cells, their secretion is increased in ER stress when ER calcium is depleted [36, 62, 67]. Secreted CDNF and MANF may function as autocrine or paracrine trophic factors, promoting cell survival. In accordance with their potential trophic activities, endogenous CDNF and MANF can be detected in human serum [72, 73]. Circulating concentrations of CDNF were not altered in PD patients while MANF concentrations were significantly increased and positively correlated with the Beck Depression Inventory scoring, which is used to measure the severity of depression. This suggests that further studies would be useful to test whether blood MANF levels can be used as a clinical marker of PD [73]. It has been proposed that serum MANF functions as a systemic regulator of inflammation and metabolic homeostasis, thus protecting against age-related deterioration [74].

### Extracellular trophic activities and plasma membrane receptors

Evidently CDNF and MANF can protect neurons as extracellular trophic factors, as demonstrated for example in animal models of PD (as discussed later in detail), and as potential intracellular regulators of protein homeostasis in the ER. Whether these two seemingly different cytoprotective activities of CDNF and MANF engage the same or different intracellular signaling pathways and molecular mechanisms is under investigation.

**Fig. 3:**
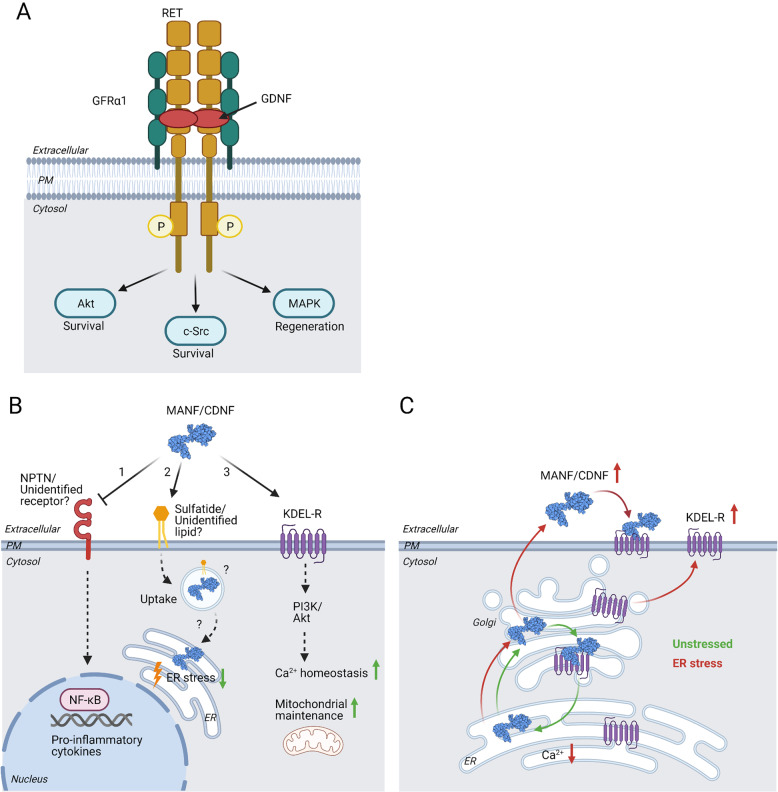
Plasma membrane receptors of GFLs and MANF/CDNF. **A** GDNF Family Ligands (GFLs): GDNF; NRTN; artemin; and persephin, function as homodimers to activate transmembrane receptor tyrosine kinase RET. Binding of GFLs to RET is mediated by GDNF family receptor-α (GFRα1–4) co-receptors, which selectively interact with the GFLs. Ligand binding leads to homodimerization and autophosphorylation of RET, resulting activation of multiple intracellular signaling cascades. GDNF binding to RET is mediated by GFRα1, and leads to activation of Akt, MAPK and c-Src pathways, promoting neuronal survival and regeneration. **B** Neuroplastin (NPTN) is a novel receptor for MANF (1). Activation of NPTN induces NF-κB transcription factor and expression of pro-inflammatory cytokines. MANF binding to NPTN decreases pro-inflammatory response and protects cells against ER stress-induced inflammation and cell death [80]. MANF binds sulfatide (2). Sulfatide is present in the plasma membrane (PM) of neurons and other cell types, suggesting that MANF can interact with sulfatide on the PM. MANF bound to sulfatide can be endocytosed to promote ER homeostasis [32]. The molecular mechanism of the potential endocytosis of MANF-sulfatide and subsequent molecular events are unclear. CDNF and MANF may bind to the KDEL-receptor (KDEL-R) on the PM via a C-terminal KDEL-like sequence (3) [36, 67]. Exogenous CDNF promoted calcium homeostasis and mitochondrial maintenance in cardiomyocytes under ER stress conditions by a mechanism dependent on its KDEL-like sequence suggesting that KDEL-R is binding CDNF [67]. The protective effect of CDNF was mediated by PI3K/Akt signaling [67]. **C** MANF, CDNF, and KDEL-R are induced by ER stress. In unstressed cells, CDNF and MANF are retained in the ER by KDEL-R, whereas in ER stress resulting from the depletion of ER calcium, they are released from cells. In ER stress, KDEL-R possibly localizes to the PM where it may bind extracellular CDNF and MANF.

In contrast to classical NTFs, publications demonstrating survival-promoting effects of extracellular CDNF and MANF on naive neurons are limited. Exogenous CDNF was able to support the development and survival of enteric DA neurons originating from enteric neural crest-derived cells in vitro [75], whereas it did not support the survival of cultured postnatal midbrain DA neurons [76]. CDNF promoted neither the survival of superior cervical ganglion (SCG) neurons, motoneurons, nor dorsal root ganglion neurons in contrast to nerve growth factor (NGF) [20]. MANF protein added to the cell culture was unable to promote the survival of naive DA or SCG neurons, in contrast to GDNF and NGF [29, 63]. Compared to naive neurons, the survival-promoting effects of CDNF and MANF have been more prominent on injured or stressed neurons [20, 29, 63]. For example, exogenous CDNF protected DA neurons against toxicity of αSyn oligomers [30]. CDNF also protected hippocampal cells against synaptotoxicity of amyloid-β peptide oligomers likely through regulation of ER stress [77]. In addition to neurons, MANF has various effects on non-neuronal cells. Exogenous MANF stimulated the proliferation of mouse and human pancreatic beta cells [56, 57, 78] that, compared many other cell types, have high physiological ER stress due to synthesis and secretion of insulin [79]. MANF also protected cultured embryonic DA neurons against ER stress and decreased induction of UPR genes via a mechanism dependent on either IRE1 or PERK pathways [63] suggesting that exogenous MANF, similarly to intracellular MANF, can promote neuron survival through regulating UPR. How could exogenous MANF regulate UPR signaling in the ER? Bai and colleagues provided one possible answer to this by proposing that extracellular MANF bound to sulfatide can be endocytosed to cells where it mediates cytoprotection by promoting ER homeostasis [32] (Fig. 3B). The molecular mechanism of the potential endocytosis of MANF-sulfatide and subsequent molecular events remain to be resolved.

Protein receptors proposed to interact with CDNF and MANF on the PM are KDEL-R and neuroplastin (NPTN) [36, 67, 80] (Fig. 3B). KDEL-R is mainly localized in the Golgi but it was also detected in the PM where it could bind CDNF and MANF through C-terminal KDEL-like sequences [36, 67] (Fig. 3B). Protective effects of exogenous CDNF against myocardial ischemia/reperfusion injury was dependent on the presence of the C-terminal lysine-threonine-glutamic acid-leucine (KTEL) sequence and PI3K-Akt signaling pathway [67]. However, the C-terminal arginine-threonine-aspartic acid-leucine (RTDL) sequence of MANF was dispensable for its neuroprotective activity in a model of cerebral ischemia [33], suggesting alternative mechanisms for exogenous MANF activity. Recently, NPTN was identified as a novel PM receptor for MANF [80] (Fig. 3B). Direct binding of MANF to NPTN decreased ER stress-mediated inflammation and cell death [80]. However, it is unclear whether NTPN is the major PM receptor for MANF. Different from MANF, CDNF does not bind NPTN or sulfatide [32, 80], suggesting that cell surface receptors for CDNF remain to be discovered.

### CDNF and MANF knockout neuronal phenotypes

There are only few studies reporting endogenous levels of CDNF in patients with PD, obviously due to limited availability of tissue material. In hippocampal samples of PD patients, CDNF levels were increased while GDNF levels were decreased suggesting that these factors could represent potential targets for modification to help attenuate cognitive decline in PD [81].

Biological functions of CDNF in the nervous system has been studied using mouse and zebrafish knockout models [75, 82, 83]. These studies indicate that CDNF expression is important for the development and maintenance of various neuronal types and circuits rather than specifically for DA neurons. Although CDNF protects midbrain DA neurons in rodent models of PD [20, 84–89], no gross anatomical changes were observed in the midbrain DA system of conventional *Cdnf*^−/−^ mice [83]. Numbers of DA neurons in the SNpc, density of tyrosine hydroxylase (TH)- or dopamine transporter (DAT)-positive fibers in the striatum, or striatal DA or DA metabolite levels did not differ between *Cdnf*^−/−^ and *Cdnf*^*+/+*^ mice [83]. However, *Cdnf* deletion did lead to changes of dopaminergic neurotransmission, as amphetamine administration induced an increased hyperlocomotor response, possibly resulting from altered function of DAT in the dopaminergic axon terminals in striatum of *Cdnf*^−/−^ mice [83]. Expression of UPR genes was not altered in the SN or striatum of *Cdnf*^−/−^ mice, suggesting that CDNF expression is not essential for the maintenance of ER protein homeostasis in the midbrain DA system [83]. Further characterization of *Cdnf*^−/−^ mice demonstrated the importance of *Cdnf* expression for the development and maintenance of neurons in the ENS. *Cdnf*^−/−^ mice suffered from an age-dependent loss of enteric neurons due to increased neurodegeneration and autophagy observed selectively in the submucosal plexus of the intestinal wall, leading to slowed gastrointestinal motility [83]. *Cdnf* expression was found to be necessary for the normal development and survival of enteric DA neurons since *Cdnf* deletion resulted in loss of DA neuronal markers in the submucosal plexus [75]. The observed ENS defect in *Cdnf*^−/−^ mice was not only for DA neurons as the numbers of NOS-, GABA-, and CGRP-expressing neurons were also decreased [75]. The data suggest that the observed functional changes in the brain dopaminergic system and loss of ENS neurons in *Cdnf*^*−/−*^ mice resemble deficiencies observed in early stage PD [83]. In a human population study, mutations in *CDNF* gene were not identified in patients with early-stage PD [90]. However, a trend towards susceptibility to PD was observed in subjects carrying an allele of an intronic *CDNF* single nucleotide polymorphism (SNP) [90].

Zebrafish *cdnf* mutants generated using CRISPR/Cas9-genome editing were viable, fertile, and had no gross morphologic phenotype [82]. Importantly, loss of *cdnf* caused impairments in dopaminergic, histaminergic, and GABAergic neurotransmitter systems in selective brain areas, indicating that CDNF is important in shaping the structure of neurotransmitter circuits in these fish CNS [82]. In the brain, *cdnf* deletion led to increased expression of *tyrosine hydroxylase* 2 which functions in DA synthesis [82]. Alterations in the neurotransmitter networks were associated with abnormal behavior, including impaired social cohesion and anxiety-related risk taking in adult *cdnf* mutants [82]. Mutant fish were also more susceptible to drug-induced seizures. Interestingly, the observed behavioral phenotypes of *cdnf* mutant fish are reminiscent of human neuropsychiatric conditions, such as schizophrenia [82], in accordance with the suggested association between a *CDNF* SNP and schizophrenia susceptibility in humans [91].

Homozygous loss-of-function mutations of the human *MANF* gene were reported as a cause of childhood diabetes, and were mechanistically connected to ER stress and impaired beta cell function [92]. A homozygous *MANF* mutation was also associated with mild intellectual disability, microcephaly, and deafness [93], suggesting that MANF has a role in brain development and normal auditory function. In accordance, *Manf* inactivation in mice resulted in a hearing loss [94]. However, characterization of conventional and CNS-specific *Manf* knockout mice indicated that endogenous MANF is not required for the maintenance of midbrain DA neurons [95]. CNS-specific deletion of *Manf* in mice did not affect the number of TH-positive DA neurons in the SNpc, number of dopaminergic fibers in the striatum, or the striatal concentrations of DA or its metabolites in adult mice [95]. Although chronic activation of UPR was detected in the brain tissue of *Manf*^−/−^ mice, it did not result in neurodegeneration [95]. In contrast to observations in *Manf* knockout mice, *Drosophila* Manf, encoded by a single homolog of human *MANF/CDNF*, appears to be essential for the maintenance of DA neurites and DA levels in the fly [96]. In *DmManf* mutant larvae, the volume of DA neurites was diminished whereas somas were maintained, suggesting that DA neurites degenerate before cell bodies [96], thus resembling degeneration of DA neurons in PD. UPR-related genes were upregulated in *DmManf* mutant embryos indicating ongoing UPR [61]. Larval lethality of *DmManf* zygotic mutants was rescued with ubiquitously expressed human *MANF* or *CDNF*, indicating that DmManf and human MANF and CDNF are functionally conserved [97]. Also in zebrafish, studies of *manf* knockdown using antisense splice-blocking morpholino oligonucleotides suggested that MANF is involved in the regulation of DA neuron development and maintenance [98]. In the *manf-1* mutant *C. elegans* worms neuronal development was normal; however, there was loss of *manf-1* activated ER stress and UPR [59, 60], resembling observations in *Manf*^*−/−*^ mice and supporting the role of MANF as a regulator of ER homeostasis.

### CDNF effects in animal models of Parkinson’s disease

**Fig. 4:**
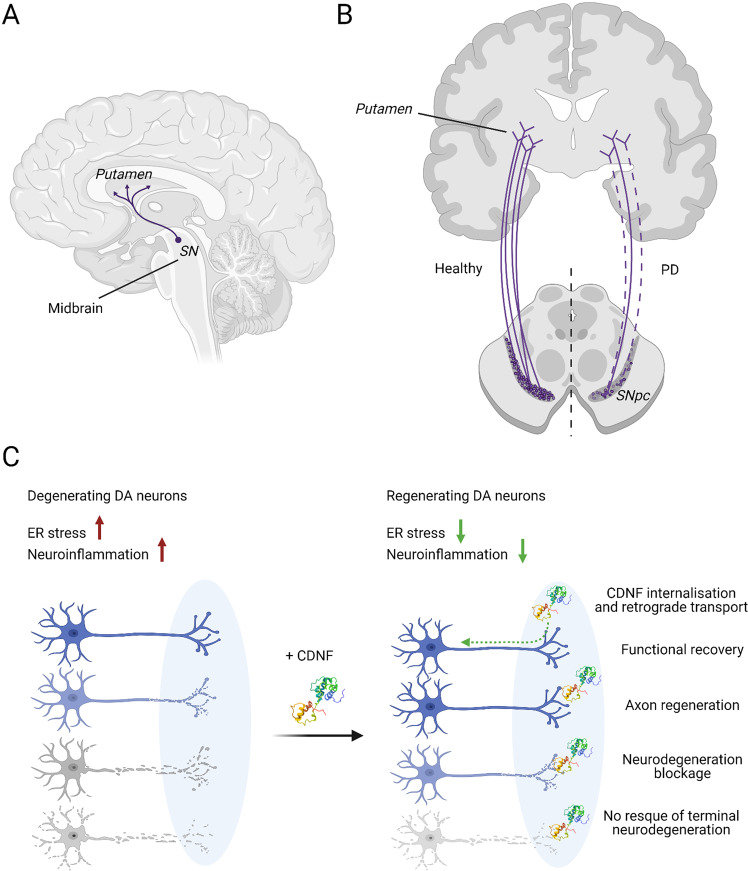
CDNF promotes survival of dopamine neurons. **A** Midbrain dopamine (DA) neurons project from substantia nigra (SN) to the putamen forming nigrostriatal pathway. **B** In Parkinson’s disease (PD), midbrain DA neurons degenerate leading to motor and non-motor symptoms. Cell bodies of DA neurons are located in the substantia nigra pars compacta (SNpc). **C** CDNF prevents neurodegeneration and induces functional recovery of injured DA neurons in animal models of PD. Therapeutic CDNF could reduce ER stress and neuroinflammation that are thought to be involved in the neuropathogenesis of PD. Differently from GDNF, CDNF does not show survival-promoting effects on naive DA neurons.

In patients with PD, DA neurons located in the SN and projecting to the striatum degenerate and die [7] (Fig. 4A, B). In animal models of PD, degeneration of DA neurons can be induced using neurotoxins 6-hydroxydopamine (6-OHDA) and 1-methyl-4-phenyl-1,2,3,6-tetrahydropyridine (MPTP) [99]. In the first in vivo study, a single injection of CDNF before the delivery of 6-OHDA into the striatum significantly reduced amphetamine-induced ipsilateral turning behavior and almost completely protected nigral DA neurons in a rat model of PD [20]. When administered 4 weeks after 6-OHDA, CDNF restored the dopaminergic function and prevented the degeneration of DA neurons at least as efficiently as GDNF [20]. In the following study, the neuroprotective effects of 2-week striatal infusions of CDNF, MANF, and GDNF were compared in a rat 6-OHDA model [84]. CDNF rescued 6-OHDA-lesioned nigral DA neurons and TH-positive fibers in the striatum, whereas MANF and GDNF had no significant effect in these measures [84]. The volume of distribution for injected MANF in the striatum was larger than that of CDNF, and both MANF and CDNF diffused significantly better than GDNF [84, 100]. Intrastriatally injected CDNF similarly to GDNF was retrogradely transported to the SN [84, 101], whereas CDNF injected to SN was not anterogradely transported to the striatum [102].

Airavaara et al. [85] demonstrated that striatal administration of CDNF was neuroprotective and neurorestorative for the TH-positive cells in the nigrostriatal DA system in a mouse MPTP model of PD. Jiaming and Niu [103] evaluated the therapeutic effects of CDNF-expressing bone marrow-derived mesenchymal stem cell (MSC) injections. Using intrastriatal, intraventricular, and intravenous routes of CDNF-MSC administration, they showed neurotrophic effects of CDNF-MSC grafts in a rat 6-OHDA model of PD by intrastriatal and intra-lateral ventricular transplantation routes. Since CDNF is mostly an intracellular protein, it was of great interest to test CDNF effects using gene therapy approaches. Bäck and colleagues [86] studied the neuroprotective effect of adeno-associated virus (AAV) serotype 2 vector expressing CDNF in a rat 6-OHDA model of PD. Elevated levels of CDNF expression in the striatum resulted in a marked decrease in amphetamine-induced ipsilateral rotations [86]. However, compared to studies using CDNF protein delivery [20, 84], gene therapy of CDNF provided only partial protection of DA neurons and their fibers [86]. One reason for this can be the retention of CDNF inside the cells with very limited diffusion of CDNF in the striatum [86]. Ren et al. [88] examined the neuroprotective and functional restorative effects of CDNF overexpression in the striatum via gene therapy with an AAV2-CDNF vector in 6-OHDA-lesioned rats. In addition to the significant restoration of TH-immunoreactive nigral neurons and striatal fibers, positron emission tomography (PET) imaging of DA transporters revealed functional recovery of the nigrostriatal DA system [88]. Compared to the study by Bäck et al. [86] the prominent neuroprotection by CDNF in the study by Ren et al. [88] may be ascribed to the optimal expression level and greater spreading of CDNF in the striatum. Hao et al. [104] demonstrated robust long-term overexpression of MANF in rat striatum using AAV9 vector-mediated gene delivery. In a 6-OHDA model of PD, intrastriatal delivery AAV9-*MANF* provided significant protection for nigral DA neurons and promoted regeneration of striatal DA fibers and increase in striatal DA levels [104]. Striatal MANF overexpression by AAV9 vector led to increased MANF levels also in the SN, suggesting that MANF was retrogradely transported from the striatum to SN, thus providing local protection for nigral neurons [104]. The ability of GDNF and related NTFs to rescue DA neurons in animal models of PD is limited when the neurotoxin-induced lesion is severe [5]. Importantly, Wang et al. [105] observed, using a rat 6-OHDA model of PD, that AAV8-CDNF administration significantly improved motor function and increased TH levels in rats with mild 6-OHDA-induced lesions, but it had limited therapeutic effects in rats with severe lesions [105]. Lentiviral vector-mediated overexpression of CDNF or MANF alone in the SN showed differential protection of dopaminergic function in the 6-OHDA model of PD [87]. While overexpression of CDNF in the SN both reduced amphetamine-induced rotational behavior and loss of striatal TH-positive innervation, overexpression of MANF in the SN only protected TH-positive cells in the nigra [87]. However, combined nigral overexpression of CDNF and MANF led to a robust reduction in amphetamine-induced rotations and protection of both DA cells and their fibers, indicating that CDNF and MANF can have synergistic neuroprotective effects [87]. Unfortunately, the levels overexpressed CDNF and MANF in the brain tissue were not reported [87], thus hampering comparisons of their neuroprotective effects. When GDNF was overexpressed in the SN it was unable to direct regeneration of TH-positive axons [106]. Since CDNF is not anterogradely transported from SN to striatum [102], its effects may resemble those of GDNF i.e., have full neuroregenerative potential only when delivered to the striatum. These data indicate that CDNF and MANF have differential modes of action and encourages using a combination of different growth factors for the treatment of PD. Indeed, an additive neurorestorative effect of CDNF and GDNF was demonstrated in the 6-OHDA model of PD in rats [89]. Experiments on cell lines and DA neurons have clearly shown that CDNF and GDNF have completely different modes of action. These additive effects observed in a rat PD model also indicated different mechanisms of action for CDNF and GDNF [89]. Both CDNF and GDNF were able to activate the survival-promoting PI3 kinase-Akt signaling pathway, but only CDNF decreased the levels of ER stress markers ATF6 and BiP, in addition to the level of phosphorylated eukaryotic initiation factor 2 α subunit (eIF2α) downstream of the UPR sensor PERK [89]. In 6-OHDA-treated PC12 cells, a cellular model of PD, CDNF treatment increased cell viability through upregulating ratio of anti-apoptotic Bcl-2/pro-apoptotic Bax proteins and downregulating caspase-3 activity, thus resembling the function of NTFs [107].

Several in vitro studies have indicated that CDNF may provide a novel therapy for neuroinflammation related to the microglia. In microglial cells, CDNF attenuated the production of pro-inflammatory cytokines prostaglandin E2 and interleukin-1β (IL-1β) as well as remarkably suppressed the phosphorylation of c-Jun N-terminal kinase (JNK) [108]. Nadella et al. [109] found that in the 6-OHDA-lesioned rats, CDNF overexpressed from a plasmid vector reduced nitrosative stress, glial markers, and IL-6 levels in the SN, but not TNFα and IL-1β levels, suggesting that CDNF may be a potential novel agent for the treatment of neuroinflammation seen in the PD.

We still have very limited information about the effects of CDNF on nigral DA neurons in NHPs. CDNF therapeutic effects were first studied in a unilateral 6-OHDA lesion model of PD in marmoset monkeys and compared with the effects of GDNF [110]. This study also monitored the severity of 6-OHDA lesions and treatment effects in vivo using ^123^I-FP-CIT (a DAT ligand) SPECT [110]. This analysis showed a significant increase of DAT binding activity in lesioned monkeys treated with CDNF, whereas no statistical difference was observed in the GDNF-treated group [110]. In a more recent study, CDNF restored SN DA neuron integrity when effects of CDNF and GDNF were compared in a rhesus monkey MPTP model of PD [111]. The animal data together demonstrate that CDNF not only protects but also restores the function of DA neurons by regulating ER stress, neuroinflammation, and counteracting cell death (Fig. 4C).

### First results of clinical trials

Since the mode of action of CDNF differs from that of GDNF, NRTN, and other growth factors tested in clinical trials for PD, and CDNF was more efficient than GDNF in protecting the function of DA neurons in animal models of PD [84, 110] it was important to test CDNF in clinic. The first clinical Phase I-II, randomized, double-blind study conducted by Herantis Pharma Plc. investigated the safety and tolerability of intermittent bilateral intraputamenal monthly infusions of CDNF (ClinicalTrials.gov Identifier: NCT03295786) [23, 112]. A two-part study in 17 patients with advanced PD was carried out in three university hospitals in Finland and Sweden. During the initial 6-month period, all patients received either placebo or CDNF at one of two dose levels. This was followed by a 6-month period, in which all patients received CDNF at one of the two dose levels, including the previous placebo group patients. Treatment was administered via a dose delivery system using intraputamenal catheters that were implanted into the putamen at the beginning of the study. Human recombinant CDNF, used in the study, was produced in a mammalian cell line and its biological activity was rigorously tested in neuronal survival assays. Intraputamenal CDNF infusions were safe and well tolerated, and thus the primary endpoint of the study was met. Exploratory endpoints included UPDRS scores and DAT PET, which was performed with a high-resolution research tomography system using DAT radioligand [^18^F]FE-PE2I [113]. A minimal clinical important difference in Unified Parkinson’s Disease Rating Scale (UPDRS III) (off) was observed in the CDNF dose-groups suggesting a potential slowing of disease progression. Furthermore, increased DAT availability in the putamen was observed with PET in some patients that received CDNF suggesting a potential improvement in dopaminergic function. Although the study of patient population with advanced PD was not designed to show efficacy of CDNF, the documented improvements in some patients were very encouraging [112].

### Concluding remarks

CDNF is an atypical neurotrophic protein that is cytoprotective both in the ER and as an extracellular factor. In addition to neuroprotective and neuroregenerative activities that, similarly to other NTFs, partially occur via the activation of PI3 kinase-Akt pathways [89, 114], CDNF also counteracts cell death by regulating UPR pathways in the ER [70, 89]. CDNF protects against toxicity of αSyn oligomers in vitro [30], and was recently shown to directly interact with αSyn, reduce propagation of αSyn aggregation and alleviate behavioral deficits induced by αSyn fibrils in mice [115]. CDNF also reduces the synthesis and release of pro-inflammatory cytokines decreasing neuroinflammation [108, 109, 114, 116]. One particularly interesting property of CDNF, which differentiates it from classical NTFs, is that its effects on naive and healthy neurons are low or even absent [20, 29, 63]. This may be very important from the clinical point of view, because it suggests a good safety profile for CDNF. Although CDNF has now been successfully tested in rodent and NHP models of PD [20, 76, 84–86, 88, 103, 109–111, 117–119], as well as in Phase I-II clinical trial in patients with PD [23, 112], several challenges remain. CDNF can regulate UPR pathways, but its receptors and signaling pathways remain poorly described. We also know very little about how CDNF acts in the ER, how it is secreted and whether it has both intracellular and plasma membrane receptors. Despite the promising results in animal models of PD, NTF- and CDNF-based treatments share a fundamental drawback; they require a direct delivery of the therapeutic protein to the brain through invasive surgery, since NTFs and CDNF cannot pass through the BBB. We have recently discovered a novel CDNF variant that acts similarly to CDNF, but can efficiently pass through the BBB [28]. Furthermore, our preliminary data show that this CDNF variant has beneficial effects in both rodent 6-OHDA and MPTP toxin models of PD when administered subcutaneously [28]. Although these data are encouraging, much more work is needed before BBB-penetrating CDNF-derived molecules can be taken to clinical trials. One important reason for the limited success of clinical development of NTFs so far is their poor pharmacokinetic characteristics, which include inability to cross tissue barriers, poor diffusion in tissues, ability to activate several receptors in different tissues and cell types, and high costs of the drug [5]. The development of small molecules selectively targeting CDNF receptors with optimized pharmacokinetic properties can open a new avenue for the development of disease-modifying treatments of neurodegenerative diseases in the future.

CDNF was also shown to have beneficial effects in animal models of Alzheimer’s disease [120], amyotrophic lateral sclerosis [121], and Huntington’s disease [122]. However, further studies are required to confirm these exciting results. Taken together, unique properties of CDNF encourage its testing in different neurological diseases, especially in those where neuronal protein homeostasis has been perturbed.
